# Repeated administration of L-alanine to mice reduces behavioural despair and increases hippocampal mammalian target of rapamycin signalling: Analysis of gender and metabolic effects

**DOI:** 10.1177/02698811251332838

**Published:** 2025-04-17

**Authors:** Abdullah Aziz, Carolina Fernandes Ferreira Alves Costa, Erying Zhao, Daniel Radford-Smith, Fay Probert, Daniel Clive Anthony, Philip William John Burnet

**Affiliations:** 1 Department of Psychiatry, University of Oxford, Oxford, UK; 2 Sainsbury Wellcome Centre, University College London, London, UK; 3 ICBAS – School of Medicine and Biomedical Sciences, University of Porto, Porto, Portugal; 4 Nephrology and Infectious Diseases R&D Group, INEB – Institute of Biomedical Engineering, i3S-Institute for Research and Innovation in Health, University of Porto, Porto, Portugal; 5 Psychological Science and Health Management Centre, Harbin Medical University, Harbin, China; 6 Deparment of Pharmacology, University of Oxford, Oxford, UK; 7 Department of Chemistry, University of Oxford, Oxford, UK

**Keywords:** Alanine, synaptic, mammalian target of rapamycin, SLC7A10, SLC1A5

## Abstract

**Background:**

The amino acid L-alanine, has been shown to be elevated in biofluids during major depression but its relevance remains unexplored.

**Aim:**

We have investigated the effects of repeated L-alanine administration on emotional behaviours and central gene expression in mice.

**Methods:**

Mice received a daily, 2-week intraperitoneal injection of either saline or L-alanine at 100 or 200 mg/kg and were exposed to the open field, light–dark box and forced swim test. The expression of L-alanine transporters (asc-1, ASCT2), glycine receptor subunits (GlyRs), NMDA receptor subunits (GluNs) mRNAs were measured, together with western blots of the signalling protein mammalian target of rapamycin (mTOR). Since L-alanine modulates glucose homeostasis, peripheral and central metabolomes were evaluated with ^1^H-NMR.

**Results:**

L-alanine administration at 100 mg/kg, but not at 200 mg/kg, to both male and female mice increased latency to float and reduced floating time in the forced swim test, but had no effect on anxious behaviour in the open field and light–dark box tests. There was a significant reduction in mRNAs encoding asc-1 and ASCT2 and GluN2B in the hippocampus of mice following 100 mg/kg L-alanine only. On western blots, hippocampal GluN2B immunoreactivity was reduced, but mTOR signalling was increased in the 100 mg/kg L-alanine group. 1H-NMR revealed gender-specific changes in the forebrain, plasma and liver metabolomes only at 200 mg/kg of L-alanine.

**Conclusions:**

Our data suggest that L-alanine may have antidepressant-like effect that may involve the modulation of glutamate neurotransmission independently of metabolism. In major depression, therefore, elevated L-alanine may be a homeostatic response to pathophysiological processes, though this will require further investigation.

## Introduction

Major depressive disorder (MDD) is a complex psychiatric illness for which conventional treatments are not always effective (McIntyre et al., 2023). Investigations of potential biomarkers for MDD to further understand its pathophysiology, permit early intervention and/or develop novel therapies, have observed an association between the disorder and the levels of the amino acid, L-alanine. Mitani and colleagues first demonstrated that elevated concentrations of plasma L-alanine in MDD correlated with symptom severity (Mitani et al., 2006). Moreover, increased urinary L-alanine was observed in both men and women with MDD (Zheng et al., 2016), which suggests that an altered concentration of the amino acid in the disorder is not influenced by gender. Other studies have demonstrated increased concentrations of peripheral L-alanine in MDD (Chen et al., 2018; Ding et al., 2014; Tian et al., 2016) and in an animal model of depression (Jianguo et al., 2019). There is also evidence for an anxiolytic effect of L-alanine when administered to rats in early life (da Silva Francisco and Guedes, 2015). However, the direct influence of L-alanine on adult emotional behaviours has not been explored. This is important to investigate as L-alanine has the potential to affect brain function through its influence on both neurotransmission and/or its involvement in metabolism.

Bidirectional transport of L-alanine across neural cell membranes is mediated by the alanine-serine-cysteine transporters, asc-1 (SLC7A10) (Helboe et al., 2003) and ASCT2 (SLC1A5) (Gliddon et al., 2009), and so changes in the central concentration of L-alanine may influence the levels of these transporters. For instance, we have shown that the application of the ASCT2 substrate, D-serine, reduced the expression of the transporter in cultured glioma cells (Sikka et al., 2010). Therefore, evaluating changes in central asc-1 and ASCT2 expression after increases in circulating L-alanine will provide some indication of the bioavailability of the amino acid to the brain, whilst the directionality of these alterations may suggest their involvement in any functional changes. In this regard, an increase in ASCT2 expression in the mouse hippocampus has been associated with depressive behaviours (Wang et al., 2017).

The asc-1 and ASCT2 transporters can also influence the function of N-methyl-D-Aspartate receptors (NMDARs) by modulating synaptic concentrations of the NMDAR co-agonist, D-serine (Billard and Freret, 2018; Foster et al., 2017). L-alanine could potentially compete with this co-agonist at asc-1 and ASCT2 sites, alter local concentrations of D-serine and change NMDAR expression and function. Alternatively, L-alanine might influence glutamate neurotransmission through its direct binding to NMDARs (Nahum-Levy et al., 2001). The influence of L-alanine on NMDAR function, however, will depend on whether it acts as an agonist or antagonist at the receptor. For instance, the pharmacological antagonism of NMDARs has antidepressant effects (Krystal et al., 2023), possibly through the alteration of the mammalian target of rapamycin (mTOR) signalling (Li et al., 2010).

L-alanine is also a full agonist of the inhibitory, strychnine-sensitive glycine receptors (GlyRs) (Lynch, 2004), of which the GlyRα2 and GlyRα3 subunits are abundant in forebrain regions (McCracken et al., 2017). An anxiolytic effect of GlyR agonists was observed in rats with a high baseline level of anxiety (McCool and Chappell, 2007), whereas stimulation of brain GlyRs has been shown to ameliorate anxiety and depressive-like behaviours associated with alcohol withdrawal (Li et al., 2019). It is possible, therefore, that L-alanine can regulate emotional behaviours by activating GlyRs in the hippocampus and cortex, which are key areas mediating the pathophysiology and treatment of MDD (Liu et al., 2017). It is worth noting that a recent study has demonstrated the expression of metabotropic glycine receptors (mGlyRs) in the pre-frontal cortex which the authors advocate as a potential therapeutic target for MDD (Laboute et al., 2023). Given that L-alanine is an agonist of GlyRs, it is reasonable to suggest that the amino acid might also bind to mGlyRs and affect brain function.

In addition to its potential function as a receptor agonist and/or modulator of amino acid transport in neural cells, L-alanine plays a significant role in glucose and metabolic homeostasis. For instance, it can be directly converted to pyruvate in the liver by alanine transaminase, which can enter the citric acid cycle for energy production (Petersen et al., 2019). Pyruvate produced from L-alanine from muscle is also used for gluconeogenesis in the liver through the Cahill cycle (Felig, 1973). The metabolic effects of L-alanine are important to consider in MDD-related research because metabolic syndromes, such as obesity and diabetes, are often comorbid (Maksyutynska et al., 2024). Indeed, the aforementioned observations that L-alanine correlated with symptom severity in MDD may indicate metabolic pathophysiological processes underlying low mood.

Based on the current literature, therefore, it is not clear whether high concentrations of circulating L-alanine seen in MDD is a homeostatic response to metabolic dysfunction in the disorder, or itself is part of the underlying pathophysiology of depression. This study aimed to explore the behavioural, molecular and metabolic effects of L-alanine administration in male and female mice. This was achieved by (1) evaluating anxious and depressive-like behaviours in mice receiving a daily, 2-week intraperitoneal injection of L-alanine at 100 or 200 mg/kg; (2) using quantitative polymerase chain reaction (QPCR) assays to measure the expression of mRNAs encoding: NMDAR subunits (GluN1, GluN2A, GluN2B), asc-1, ASCT2, GlyRα2 and GlyRα3 in the hippocampus and cortex of mice following behavioural tests; (3) employing western blots to determine the levels of phopho-mTOR/total mTOR ratios, which indicates activation of mTOR signalling (Liu et al., 2015), and other proteins encoded by the transcripts affected by L-alanine administration and (4) examine the forebrain (cortex and hippocampus), plasma and liver metabolomes using 1H-NMR.

## Materials and methods

### Animals

All procedures were carried out in accordance with the UK Animals (Scientific Procedures) Act 1986 and were approved by the Animal Welfare and Ethical Review Body of the University of Oxford. Male and female, 6-week old, CD1 mice were purchased from Envigo, UK, and were kept in standard laboratory housing conditions (12 h light–dark cycle, lights on at 7 a.m.; 21 ± 1°C; humidity 50% ± 5%) with access to both food and water ad libitum.

### Experimental design

#### L-alanine administration

Mice received a daily, intraperitoneal injection of either saline, L-alanine (100 mg/kg in saline) or L-alanine (200 mg/kg in saline) for 2 weeks, prior to the start of behavioural tests. Previous work has shown that an L-alanine intraperitoneal injection at 200 mg/kg to mice initiates a metabolic response (Adachi et al., 2018). We also tested the effect of 100 mg/kg L-alanine since an earlier study showed altered glucose metabolism in mice fed with L-alanine at less than 200 mg/kg (Gray et al., 2007). All animals were monitored for adverse effects of L-alanine. Some of the male mice had to be removed from the experiment as they had sustained severe wounds from fighting.

#### Behavioural tests

All behavioural testing was carried out during the light phase. The open field test, light–dark box and forced swim test were performed in sequence as previously reported (Chan et al., 2023).

*Open field:* This test was used to assess both anxious and exploratory behaviour. Animals were placed in the corner and allowed to explore the apparatus for 5 min. Parameters measured include time spent in the centre of the field and the number of line breaks (entries into the centre zone).

*Light–dark box:* The box consists of a small dark safe compartment (one-third) and a large illuminated aversive compartment (two-thirds), separated by a partition wall that had a small opening. The mouse was placed in the dark compartment and was allowed to freely explore the box for 5 min. The time spent in the light box, latency to enter the light and number of entries into the light side were measured. The box was cleaned thoroughly before each test.

*Forced swim test:* The purpose of this test was to evaluate behavioural despair in mice and was chosen to allow comparisons with studies that used the same test to examine traditional antidepressant drugs. The mouse was placed in a transparent container filled with 15 cm of water to swim for 5 min. Water was changed between each animal to remove odours and the temperature was adjusted to 30°C. Time immobile between 3 and 5 min and the latency to first immobility over the entire duration (5 min) were measured as previously described (Chan et al., 2023). Immobility was defined as a total absence of movement except slight motions to balance the body.

### Transcript and protein measurements

#### Tissue collection

All animals were anaesthetised under isoflurane gas, 24 h after the last injection of L-alanine. This time point was chosen to explore the sustained, rather than transient (Adachi et al., 2018), effects of L-alanine administration. After checking for the absence of a pedal reflex, cardiac puncture was used to collect whole blood in ethylenediaminetetraacetic acid (EDTA, 0.1 M, pH8)-coated tubes and centrifuged after 30 min at RT (10 min, 2000 × *g*). Tissue was transcardially perfused with heparinised saline until the liver was clear of blood. Brains were removed, and the frontal cortex and hippocampus were dissected and stored at −80°C. These brain areas were identified as being key regions involved in the modulation of emotional behaviours (Liu et al., 2017). Fragments of the liver were also collected and stored at −80°C prior to analysis.

#### RNA extraction and quantitative PCR (QPCR)

Total RNA from all tissues was isolated using the Tri Reagent (Thermo Fisher Scientific, UK) and following the manufacturer’s instructions. The purified RNA was dissolved in RNase-free water and the concentration was determined using a NanoDrop™-ND 1000 UV-Vis Spectrophotometer (Labtech, UK). All RNA samples (1 µg) were DNase (Promega) treated and subjected to reverse transcription (RT) using the High Capacity cDNA RT kit (Thermo Fisher Scientific, UK). A negative RT reaction was performed with 1 μg of total RNA without reverse transcriptase to check for genomic DNA contamination in the QPCR. The QPCR assays were performed in triplicate, in a total volume of 12 µl, which included 6 µl of 2× PowerTrack SYBR Green Master Mix (Applied Biosystems™, Thermo Fisher Scientific, UK), 1 µl each of forward and reverse specific primers (10 pmol/µl) and 4 µl RT preparation. The primer sequences used were as follows: asc-1, (forward: 5′-CTTGCACCATCATCATCGGG-3′; reverse: 5′-AGAGCAGTAGGAATCCAGCC-3′); ASCT2, (forward: 5′-GCTTGGTCGTGTTCGCTATA-3′; reverse: 5′-GCCAGTCCACGGCCAAGATC-3′); GluN1, (forward: 5′-ATCATCCTGCTGGTCAGCGA-3′; reverse: 5′-AGCAGAGCCGTCACATTCTT-3′); GluN2A, (forward: 5′-GCTTTCCTTGAACCCTTCAG-3′; reverse: 5′-AACTTAGCCAAAGGGAAAGCTCCCGA-3′); GluN2B, (forward: 5′-TTGGTGAGGTGGTCATGAAG-3′; reverse: 5′-ACCTTCTGCCTTCTTAGAGCC-3′); mGlyR, (forward: 5′-CTTTACTCTTCTGCCTCCTGCT-3′; reverse: 5′-AGTCCCCAGTGTAGAGGTAAGA-3′); GlyRα2, (forward: 5′-CTGCAAAGACCATGACTCCAGG-3′; reverse: 5′-TCGGTAGTCCATGGTGGTTTCT-3′); GlyRα3, (forward: 5′-TTCTGGGAAGCCGCACTGTTAC-3′; reverse: 5′-ATGGAGCCAAAGCTGTTTATGA-3′); and glyceraldehyde phosphate dehydrogenase (GAPDH) (forward: 5′-GTATTGGGCGCCTGGTCACC-3′; reverse: 5′-CGCTCCTGGAAGATGGTGATGG-3′). Expression levels were calculated relative to GAPDH expression and fold changes were calculated using the ΔΔCt method (Livak and Schmittgen, 2001).

#### Western blotting

Immunoblotting was carried out as previously described (Burnet et al., 2011), on hippocampus and frontal cortex tissues from four male and four female mice of each group. Briefly, brain tissues were homogenised in RIPA buffer (Sigma-Aldrich, UK) containing a 0.1% (v/v) protease inhibitor cocktail. Protein concentrations of tissue homogenates were determined using Bradford’s protein assay (Bio-Rad Laboratories, CA, USA). Total protein (40 μg) was loaded into a precast AnyKD Mini-Protean TGX Gel (Bio-Rad) and separated with SDS-PAGE in 1 × Running Buffer (25 mM Tris base, 250 mM Glycine, 0.1% SDS, dH2O). Proteins were then transferred to a polyvinylidene difluoride (PVDF) membrane overnight using electroblotting in 1 × Transfer Buffer (25 mM Tris base, 192 mM Glycine, 20% Methanol, dH2O). The membrane blots were reactivated in methanol and incubated for 1 h at room temperature in a blocking buffer (0.1% PBS-Tween20 (PBS-T) with 5% skim milk). Primary and secondary antibodies were prepared in 0.1% PBS-T with 2% milk and added to blots sequentially for 1 h and 40 min, respectively, separated by three 20 min washes in Tris-buffered saline with PBS-T. Antibodies used in western blots included anti-GluN2B (1:1000, Merck Life Science UK Ltd), anti-[phospho-Ser2448]mTOR (1:1000, Thermo Fisher Scientific, UK), anti-mTOR (1:1000, Thermo Fisher Scientific, UK) and anti-β-Actin (1:5000, Merck Life Science UK Ltd, UK). Horseradish peroxidase-conjugated goat anti-rabbit (1:10,000, Thermo Fisher Scientific, UK) and goat anti-mouse (1:5000, Thermo Fisher Scientific, UK) were used as secondary antibodies. Immunoreactivity was visualised with enhanced chemiluminescence (ECL^TM^ Prime Western Blotting System, Merck Life Science UK Ltd, UK) and exposure to Hyperfilm^TM^ ECL^TM^ (Merck Life Science UK Ltd, UK) at various time points. Densitometric analysis of immunoreactive bands was performed using AlphaImager 3400 to quantify relative protein expression using film exposures below the saturation point. β-Actin was used as an internal control to normalise the optical density values in each sample.

### ^1^H NMR metabolomics

#### Sample processing

Metabolites were extracted from the forebrain (pooled hippocampus and frontal cortex), plasma and liver samples as previously described (Radford-Smith et al., 2022). Briefly, fresh frozen tissue 100 mg (±10%) was immersed in 800 µl ice-cold acetonitrile (50%) in a 2 ml Eppendorf tube, and homogenised in a bead-beater (TissueLyser LT, Qiagen, Hilden, German) at 40 cycles/s for 2 mins. Samples were then centrifuged at ~11,000 × *g* for 5 min at 4°C. Supernatants were snap-frozen on dry ice, lyophilised overnight and stored at −80°C until the day of NMR. When ready for analysis, lyophilised samples were placed at room temperature and resuspended in 550 µL of NMR buffer (75 mM sodium phosphate buffer prepared in D_2_O, pH 7.4). The plasma samples were also allowed to thaw at RT and 150 µL was added to 400 µL NMR buffer.

#### ^1^H NMR experiments

All prepared samples were then placed in a 5 mm NMR tube and measured using a 700-MHz Bruker AVII spectrometer operating at 16.4 T, equipped with a 1H (13C/15N) TCI cryoprobe, as described previously (Radford-Smith et al., 2022). Plasma and liver spectra were acquired using a spin-echo sequence (Carr-Purcell-Meiboom-Gill), 32 data collections, an acquisition time of 1.5 s, a relaxation delay of 2 s, and a fixed receiver gain. Brain spectra were acquired with a Nuclear Overhauser Effect Spectroscopy sequence as previously described (Radford-Smith et al., 2022). All samples were run within 9-h of being thawed.

#### ^1^H NMR data processing

Spectra were phased, baseline-corrected and referenced to the lactate doublet at δ = 1.33 ppm in Topspin 4.1.4 (Bruker). ACD/Labs Spectrus Processor Academic Edition 12.01 (Advanced Chemistry Development, Inc., Toronto, Canada) was used to manually bin each resonance signal, excluding all noise from the analysis. The integrals of these bins were sum normalised and exported to R. Metabolites assignment was conducted through a mix of in-house databases, literature reviews and 2D total correlation spectroscopy (TOCSY) experiments.

### Statistical analysis

Statistical analysis of all data was performed using SPSS (version 25, IBM Corp., Armonk, NY, USA). Skewness of data was explored with the Shapiro–Wilkes test, and identified outliers (⩾3SD) were removed prior to analysis. All results are presented as mean ± standard error of the mean (SEM).

*Behavioural and mRNA expression data:* Multivariate General Linear Models were used to assess group (saline, L-alanine (100 mg/kg), L-alanine (200 mg/kg)) × gender (male, female) interactions and main effects of group. A significant interaction (*p* < 0.05) or main effect of the group was followed up with pairwise comparisons and Bonferroni corrections.

*Western blot data:* One-way ANOVA were performed with post hoc Tukey’s tests for multiple comparisons, if there was a significant overall effect (*p* < 0.05).

^1^*H NMR data:* The ROPLS package in R was used for principal component analysis (PCA) of the brain, blood and liver metabolomes to determine whether there were any between-group differences. PCA score plots were visually inspected for group differences across each principal component.

## Results

### Effect of L-alanine on emotional behaviours

**Figure 1.**
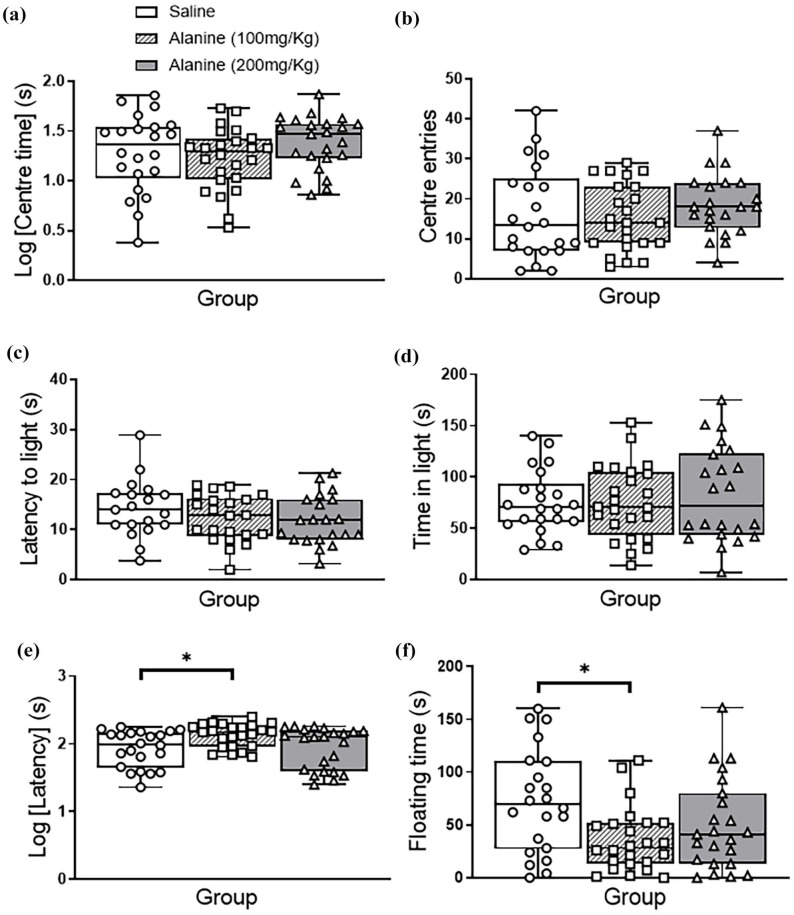
Emotional behaviours of male and female mice following a daily, 2-week administration of saline, 100 mg/kg L-alanine or 200 mg/kg L-alanine. (a and b) In the open field test, L-alanine did not affect time in the centre or centre entries. (c and d) In the light–dark box test, there was no effect of L-alanine administration on latency to leave the dark area or time spent in the light compartment. (e) In the forced swim test, there was a significant overall effect of the group on the latency for mice to float, with pairwise comparisons revealing that the 100 mg/kg L-alanine group displayed a greater latency to float compared to controls. (f) In the forced swim test, there was a significant overall effect of the group on the time mice spent floating, with pairwise comparisons revealing that the 100 mg/kg L-alanine group spent less time floating than controls. There were no group × gender interactions observed (F_1,60_ < 3.0, *p* > 0.05). Data are presented as a median and interquartile range and analysed with two-way ANOVA. *p < 0.05; *n* = 10–12 mice/gender; *n* = 20–24 mice/group

The administration of L-alanine to mice at two concentrations (100 and 200 mg/kg) compared to saline injections, did not affect time spent in the central area and distance travelled in the open field test (F_2, 62_ < 3.0, *p* > 0.05; Figure 1(a) and (b)), or time spent in the light area and latency to enter the light area in the light–dark box test (F_2, 62_ < 3.0, *p* > 0.05; Figure 1(c) and (d)). No group × gender interactions were observed for each test (F_1, 60_ < 3.0, *p* > 0.05). In the forced swim test, there was a significant effect of group on latency to float (F_2, 62_ = 4.09, *p* = 0.022; Figure 1(e)) and floating time (F_2, 62_ = 4.45, *p* = 0.016; Figure 1(f)). Pairwise comparisons revealed that only 100 mg/kg L-alanine increased latency to float (*p* = 0.049) and reduced floating time (*p* = 0.013). There were no group × gender interactions detected in any of the measured parameters of the forced swim test (F_1, 60_ < 3.0, *p* > 0.05).

### Gender-independent effects of L-alanine on the expression of glutamate-related genes

**Figure 2.**
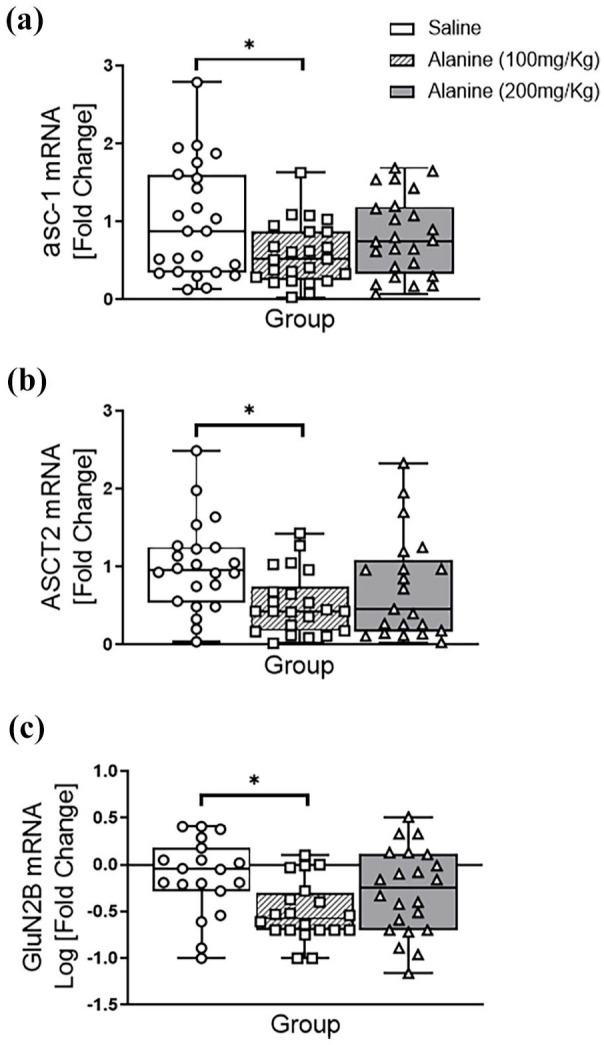
Expression of L-alanine transporters (asc-1, ASCT2) and GluN2B NMDAR subunit mRNAs in the hippocampus of male and female mice following a daily, 2-week administration of saline, 100 mg/kg L-alanine or 200 mg/kg L-alanine. For each gene, there was an overall effect of group, with pairwise comparisons revealing a significant reduction of mRNAs encoding (a) asc-1; (b) ASCT2 and (c) Glun2B compared to controls. There were no group × gender interactions observed (F_1,60_ < 3.0, *p* > 0.05). Data are presented as a median and interquartile range from fold changes compared to saline-administered mice and analysed with two-way ANOVA. *p < 0.05; *n* = 10–12 mice/gender; *n* = 20–24 mice/group.

In the hippocampus, there was a significant overall effect of L-alanine on the expression of mRNAs encoding asc-1 (F_2, 62_ = 5.85, *p* = 0.005; Figure 2(a)), ASCT2 (F_2, 62_ = 3.28, *p* = 0.048; Figure 2(b)), and GluN2B (F_2, 62_ = 5.21, *p* = 0.010; Figure 2(c)). Pairwise comparisons demonstrated that only the 100 mg/kg administration significantly reduced the expression of these genes (*p* < 0.05). No significant changes were observed for GluN1 mRNA (F_2, 62_ < 3.0, *p* > 0.05) or GluN2A (see Table 1) following L-alanine administration.

**Table 1.** Effect of a daily, 2-week administration of saline, 100 mg/kg L-alanine or 200 mg/kg L-alanine on hippocampal and cortical gene expression in male and female mice. Data are expressed as mean ± SEM of fold changes relative to saline controls and analysed with Multivariate General Linear Models and post hoc pairwise comparisons.

Brain area/gene	mRNA fold change (±SEM)		
	Saline	Alanine (100 mg/kg)	Alanine (200 mg/kg)
Hippocampus	*n* = 20	*n* = 23	*n* = 22
GluN1	1.00 (0.10)	1.01 (0.08)	1.06 (0.08)
GluN2A	1.00 (0.16)	1.11 (0.21)	1.29 (0.3)
mGlyR	1.00 (0.27)	0.69 (0.10)	0.59 (0.09)
GlyRα2	1.00 (0.18)	0.84 (0.19)	1.12 (0.28)
GlyRα3	1.00 (0.14)	0.79 (0.14)	0.76 (0.18)
Frontal cortex	*n* = 20	*n* = 23	*n* = 22
ASCT2	1.00 (0.17)	1.18 (0.21)	1.00 (0.19)
GluN1	1.00 (0.25)	0.59 (0.08)	0.95(0.18)
GluN2A	1.00 (0.19)	0.57 (0.15)	0.30 (0.09)
GluN2B	1.00 (0.19)	0.64 (0.13)	0.42 (0.09)
mGlyR	1.00 (0.14)	0.85 (0.14)	0.80 (0.10)
GlyRα2 (total)	1.00 (0.13)	1.47 (0.32)*	0.66 (0.08)
GlyRα2 (female)	1.00 (0.18)	2.11 (0.20)^†^	0.78 (0.23)
GlyRα2 (male)	1.00 (0.20)	0.82 (0.19)	0.64 (0.19)
GlyRα3 (total)	1.00 (0.16)	1.98 (0.40)*	0.82 (0.18)
GlyRα3 (female)	1.00 (0.29)	3.14 (0.33)^†^	0.74 (0.37)
GlyRα3 (male)	1.00 (0.33)	0.79 (0.71)	0.69 (0.31)

GluN1, GluN2A, GluN2B: NMDAR subunits; mGlyR: metabotropic glycine receptor; GlyRα2, GlyRα3: ionotropic glycine receptor subtypes.

* *p* < 0.05, compared to saline (overall effect of the group).

† *p* < 0.05, compared to saline (group × gender interaction, pairwise comparisons).

**Figure 3.**
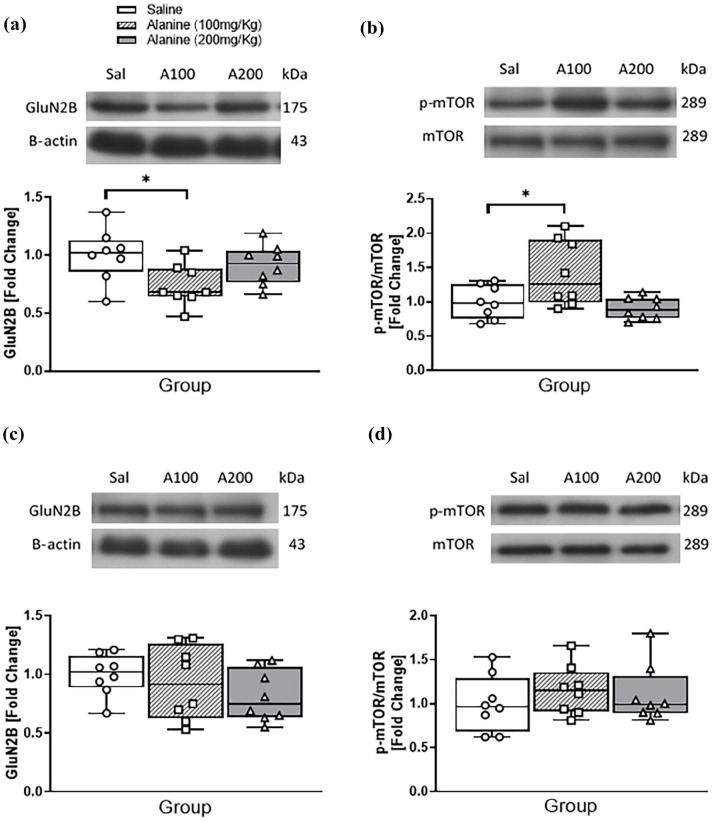
Western blot analysis of GluN2B and (phospho-Ser2448)-mTOR/mTOR ratios in the hippocampus (a and b) and cortex (c and d) of male and female mice following a daily, 2-week administration of saline, 100 mg/kg L-alanine or 200 mg/kg L-alanine. (a) Top panel: representative chemiluminescent images of GluN2B and β-actin immunoreactivity in hippocampal extracts from each group. Bottom panel: Densitometric quantification of immunoblots expressed as fold change of GluN2B/β-actin ratios compared to saline controls. (b) Top panel: representative chemiluminescent images of (phospho-Ser2448)-mTOR (p-mTOR) and total mTOR immunoreactivity in hippocampal extracts from each group. Bottom panel: Densitometric quantification of immunoblots expressed as fold change of p-mTOR/mTOR ratios compared to saline controls. (c) Top panel: representative chemiluminescent images of GluN2B and β-actin immunoreactivity in hippocampal extracts from each group. Bottom panel: Densitometric quantification of immunoblots expressed as fold change of GluN2B/β-actin ratios compared to saline controls. (d) Top panel: representative chemiluminescent images of (phospho-Ser2448)-mTOR (p-mTOR) and total mTOR immunoreactivity in hippocampal extracts from each group. Bottom panel: Densitometric quantification of immunoblots expressed as fold change of p-mTOR/mTOR ratios compared to saline controls. Data are presented as median and interquartile range and analysed with one-way ANOVA. kDa: molecular weight of each protein; Sal: saline, A100: L-alanine (100 mg/kg); A200: L-alanine (200 mg/kg). *p < 0.05, compared to controls; 8 mice/group.

Western blots were performed to confirm that changes in GluN2B mRNA affected encoded protein and mTOR signalling. One-way ANOVA of densiometric data from immunoblots demonstrated an overall effect of L-alanine on immunoreactivities for GluN2B (F_2, 21_ = 3.85, *p* = 0.038; Figure 3(a)) and [phosphor-Ser2448]-mTOR/mTOR (F_2, 21_ = 4.50, *p* = 0.024; Figure 3(b)). Tukey post hoc analysis revealed that compared to controls, only 100 mg/kg reduced the levels of GluN2B (*p* = 0.033), but elevated the levels of [phosphor-Ser2448]-mTOR/mTOR. There were no gender-independent effects of L-alanine administration on gene expression in the frontal cortex. Western blots also showed that L-alanine did not affect cortical GluN2B (Figure 3(c)) or phospho-mTOR (Figure 3(d)) levels.

### Effects of L-alanine on additional hippocampal and cortical gene expression

The analysis of genes measured in the hippocampus and cortex following the administration of L-alanine is summarised in Table 1. In the hippocampus, a 2-week, daily administration of L-alanine at 100 or 200 mg/kg, did not alter the expression of BDNF, GluN1, Glun2A, mGlyR, GlyRα2 or GlyRα3 mRNAs. However, in the frontal cortex, there was an overall effect of group for GlyRα2 (F_1, 60_ = 7.22, *p* = 0.002) and GlyRα3 (F_1, 60_ = 8.06, *p* = 0.001), and a significant group × gender interaction for GlyRα2 (F_1, 60_ = 5.48, *p* = 0.008) and GlyRα3 (F_1, 60_ = 8.13, *p* = 0.001). Pairwise comparisons demonstrated that 100 mg/kg increased the expression of these genes in female mice only (see Table 1 for gender-specific changes). The administration of L-alanine did not alter the expression of Asc-1, ASCT2, BDNF, GluN1 or mGlyR in the frontal cortex.

### Forebrain, plasma and liver metabolomes 24 h after the last injection of L-alanine or saline

**Figure 4.**
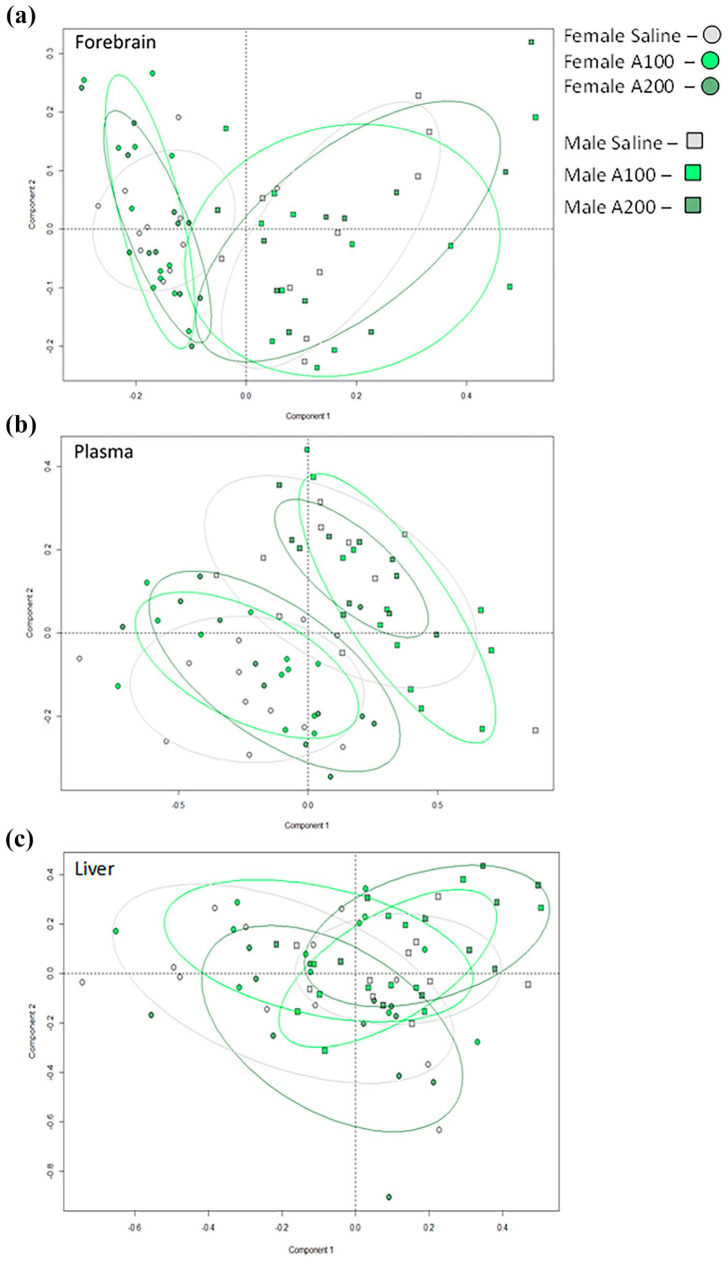
PCA plots of all metabolites in brain and peripheral tissue extracts by ^1^H NMR spectroscopy. Unsupervised analysis of all data revealed that gender was driving an observed separation of data points in (a) the forebrain; (b) plasma and (c) the liver. No effect of treatment was detected. *n* = 10–12 mice/gender; *n* = 20–24 mice/group.

Analysis of the metabolomic data from aqueous NMR spectra using unsupervised PCA revealed gender-specific profiles in all tissue types, but no obvious separation by treatment groups (Figure 4). There were no detectable changes in L-alanine concentrations and associated metabolites (pyruvate, lactate, glucose) or metabolites related to glutamate signalling (glutamate, D-serine, glycine) in all tissues from L-alanine compared to saline-administered groups.

## Discussion

We have demonstrated that a daily, repeated injection of 100 mg/kg, but not 200 mg/kg of L-alanine to mice, attenuated behavioural despair in the forced swim test without affecting anxious behaviours in the open-field test and the light–dark box. An earlier study has shown that the antidepressant fluoxetine, a serotonin reuptake inhibitor, also has differential effects on anxiety- and depression-like behaviours in mice and that these effects are lost at a high dose of the drug (Dulawa et al., 2004). Furthermore, our study revealed an elevated level of mTOR signalling in the hippocampus at the low dose of L-alanine together with a reduction of hippocampal NMDARs containing GluN2B subtypes. An examination of brain, plasma and liver metabolomes with ^1^H-NMR revealed that the behavioural effect of L-alanine was independent of its metabolic role. The interpretation and implications of our findings are considered below.

The present investigation was based on the observation that circulating concentrations of L-alanine correlated with symptom severity in MDD (Mitani et al., 2006). Our data suggest that changes in L-alanine in the disorder may be a homeostatic response to attenuate the pathophysiological changes associated with and/or underlying MDD. The molecular profile we have demonstrated at 100 mg/kg L-alanine administration paralleled those that have been reported for a conventional antidepressant. Fluoxetine has been shown to elevate brain (phospho-Ser 2448)-mTOR/mTOR ratios in mice (Liu et al., 2015), which is indicative of increased mTOR signalling. Activation of the mTOR pathway is also observed following the administration of ketamine, a rapidly acting antidepressant (Kato, 2023; Li et al., 2010). However, since the modulation of mTOR has been linked to both ASCT2 (Fuchs et al., 2007) and NMDAR function (Chan et al., 2017), it is important to consider the potential mechanisms through which each of these genes may have affected mTOR and alleviated behavioural despair in the current investigation.

A reduction in asc-1 and ASCT2 gene expression in the hippocampus after L-alanine administration may have been a response to hinder an elevation of intracellular concentrations of the amino acid so that neural cell metabolism is maintained. The decreased function of these transporters may have then led to increased synaptic concentrations of D-serine, which also has the potential to impart an antidepressant effect (Chan et al., 2017). In support of this supposition is an investigation reporting that, in contrast to our findings, elevated ASCT2 expression and reduced D-serine concentrations in the hippocampus of mice were associated with depressive-like behaviours (Wang et al., 2017). A failure to detect increased brain D-serine concentrations in our metabolomic experiments may have been because overall levels of D-serine remained constant but its distribution between extracellular and intracellular compartments changed (Sikka et al., 2010), particularly if L-alanine was also competing with D-serine for intracellular transport. However, the metabolomic measures were in pooled samples of the hippocampus and cortex, and since ASCT2 mRNA did not change in the frontal cortex, it is likely that any alteration in hippocampal D-serine was ‘diluted’ and obscured. Although elevation of synaptic D-serine by L-alanine is an intriguing possibility, it is not fully supported by our NMDAR gene expression data.

Studies have shown that GluN2A-NMDARs have a high affinity for D-serine compared to GluN2B-NMDARs, which preferentially use glycine as a co-agonist (Madry et al., 2007). Therefore, a speculative elevation of hippocampal D-serine following L-alanine administration may have been expected to alter GluN2A, rather than GluN2B subunits, as we have previously observed in the mouse cerebellum (Burnet et al., 2011). However, perhaps more importantly, earlier work has demonstrated that L-alanine has an affinity for NMDARs and can act in a similar way to glycine itself. That is, the application of L-alanine to neuronal cultures from the hippocampus in the presence of glutamate, elicits NMDAR-mediated currents that can be reduced with saturating concentrations of the amino acid or glutamate (Nahum-Levy et al., 2001). The desensitisation of NMDAR function by L-alanine might be reflected in our observed decrease of hippocampal GluN2B-NMDARs. In this instance, continued desensitisation of NMDARs by repeated L-alanine administration led to a decrease in the production of the receptor to counter-balance elevated co-agonist binding. The unaltered expression of GluN1, the proposed binding site of L-alanine, may confound this theory, though stable levels of GluN1 in the presence of changes in GluN2 subunits have precedents (Acutain et al., 2021). Reduced GluN2B expression is consistent with the antidepressant effect of fluoxetine and ketamine being associated with the inhibition of GluN2B-containing NMDARs, through their direct antagonism (Kiss et al., 2012; Miller et al., 2014). Specific GluN2B antagonists have also been shown to have antidepressant effects in humans (Preskorn et al., 2008).

In view of our findings, we tentatively propose that in our study, L-alanine acted like glycine and bound to extra-synaptic/interneuron NMDARs containing GluN2B subunits (Chan et al., 2017), which desensitised and reduced in expression. A decrease in GluN2B-NMDAR signalling then led to elevated mTOR signalling (Autry et al., 2011), which is consistent with the elevation in phospho-mTOR protein observed in the current investigation (Figure 4). An additional consequence of elevated hippocampal L-alanine may have been an increase in extracellular D-serine through a reduction in its cellular uptake via asc-1 and ASCT2 transporters, which may have led to elevated synaptic GluN2A-NMDAR signalling. However, we did not observe an elevation in brain D-serine concentrations, possibly for the reasons discussed above, and/or like L-alanine, there was a transient change in its levels which occurred long before tissues were harvested (see below). Until proven, therefore, the notion that D-serine mediated the molecular effects of L-alanine administration remains speculative. Notwithstanding the aforementioned considerations, it is also important to note that theories of NMDAR-based mechanisms of antidepressant action are not limited to the hippocampus, and several studies suggest cortical areas play key roles (Liu et al., 2017). The fewer molecular changes in the frontal cortex at the L-alanine concentration that had an antidepressant effect might be linked to L-alanine availability in this area.

The abundance of asc-1 and ASCT2 mRNAs in the current investigation could be used as proxy markers for the presence of central L-alanine. Our data from mice that received 100 mg/kg L-alanine, therefore, suggests that hippocampal L-alanine is at a sufficient concentration after exogenous administration to elicit a response in asc-1 and ASCT2 expression, whereas in the cortex, L-alanine levels may be too low to do this and/or cortical asc-1 and ASCT2 are less sensitive to changes in the concentration of their substrates. Indeed, unaltered levels of GluN2B in the cortex may also suggest that the availability of L-alanine is less than that in the hippocampus. Arguing against exogenous L-alanine not reaching the cortex might be the observed elevation of cortical GlyRα2 and GlyRα3 expression in female mice. That is, since L-alanine is an agonist at these receptors (Lynch, 2004), their elevation could be in response to, and thus indicate, the presence of the amino acid. However, this does not explain the lack of effect of L-alanine in the cortex of male mice. Further investigations are required to explore the neuroanatomical specific modulation of asc-1 and ASCT2 by L-alanine which may involve, discerning their potential differential properties on synapses and glial in the hippocampus and cortex, and an examination of blood–brain barrier modulation of L-alanine uptake in these brain areas. The functional consequences of increased GlyRs in the female cortex by L-alanine also warrant further investigation, though it would seem that these changes are not involved in the antidepressant effect of the amino acid.

The most unexpected finding of the present study was that L-alanine administered at 200 mg/kg did not reduce immobility in the forced swim test, unlike the lower dose. A reduction in behavioural despair following an apparent ‘U-shaped’ dose response to pharmacological agents is not unusual and has been reported for fluoxetine (Dulawa et al., 2004) and other compounds with antidepressant actions. For instance, Fitzgerald and colleagues demonstrated that several cholinesterase inhibitors exhibited U-shaped dose–response curves in mice exposed to the forced swim test (Fitzgerald et al., 2021). In the latter study, it was suggested that higher concentrations of the inhibitors affected other neural pathways and reduced the availability of the compounds to the antidepressant circuitry. Similarly, since L-alanine is metabolically active, the unaltered levels of brain GluN2B, asc-1 and ASCT2 expression at high concentrations of L-alanine in the current study might suggest that the amino acid was not available to interact with these genes because it was diverted for other functions and/or metabolised.

Inspection of forebrain, plasma and liver metabolomes of L-alanine compared to saline-injected mice did not reveal changes in small molecules that are directly associated with L-alanine metabolism. An effect of gender on these metabolomes is not consistent with the gender-independent action of L-alanine on behaviour, which further suggests that an antidepressant action of the amino acid is not associated with metabolism. A key reaction for L-alanine utilisation is its conversion to pyruvate by alanine transaminase (Petersen et al., 2019), and this can be then converted to lactate, enter the citric acid cycle which produces energy and mediates the synthesis of other amino acids, and/or is used for the synthesis of glucose by the Cahill cycle (Felig, 1973). It is reasonable to assume, therefore that pyruvate, lactate and/or L-alanine itself would be elevated following administration of the amino acid, particularly at the higher dose. However, in the current study, tissues were collected 24 h after the last L-alanine administration, and so it is likely that any metabolic signatures resulting from this injection had dissipated by the time mice were culled. Studies have shown a relatively short half-life (30–40 min) of L-alanine administered to humans (Elia et al., 1980) and mice (Wood et al., 2021). Thus, since metabolomic analyses were not performed after an acute injection of L-alanine or at time points soon after its administration, information on the fate of L-alanine and associated metabolites in our investigation is limited.

Our current metabolic data, therefore, demonstrate that any immediate effects of L-alanine on glucose metabolism are not sustained, though their influence on the observed molecular and behavioural changes cannot be ruled out. Parenthetically, L-alanine has been shown to attenuate metabolic dysfunction in mice (Dandare et al., 2021), and its potential as a therapeutic agent for metabolic disease in humans is currently being investigated (see ClinicalTrial.gov: NCT06419686). This further suggests that the administration of L-alanine has long-term physiological benefits, in spite of its short half-life. Future studies are required to examine the central and peripheral metabolomes of a single dose of L-alanine, not only to understand the mechanisms underlying the antidepressant effect of 100 mg/kg of L-alanine but also to explain the null effect of 200 mg/kg, for example, whether high doses of L-alanine activate metabolic pathways that would reduce its availability to the brain. Another limitation of the study is that only one test of behavioural despair was used. Other tests such as tail-suspension and sucrose preference, together with additional assessments of anxiety, should be applied to confirm that L-alanine modulates depressive-like behaviours only. Finally, our proposed mechanisms underlying the L-alanine antidepressant effect in the current investigation assume that the changes in transcript and protein levels of the encoding genes measured translate to function. That is, a reduction in hippocampal GluN2B levels is presumed to reflect a decrease in Glun2B-containing NMDAR function and thence behaviour. This would require corroboration with, for example, electrophysiological studies to demonstrate L-alanine-mediated alteration in hippocampal NMDAR responses in vivo, which are relevant to emotional behaviours. In this regard, our observed changes in gene expression may have been local homeostatic responses to prevent L-alanine changing hippocampal function and that the antidepressant effect is mediated elsewhere through other systems influenced by the amino acid.

In conclusion, we have provided evidence for an antidepressant-like action of L-alanine in mice, which may help understand the observed increase in circulating L-alanine levels in MDD. Thus, elevated L-alanine in MDD, which correlates with symptom severity, may be a homeostatic response to the pathophysiological processes that underlie mood dysfunction, rather than the amino acid itself having a pathophysiological role. However, the L-alanine response in MDD fails to mitigate depressive symptoms, perhaps because excessive circulating concentrations of the amino acid are more readily metabolised, thus reducing its availability to impart any antidepressant action. This is consistent with our finding that a high dose of L-alanine does not affect behavioural despair as seen with a lower dose. Our metabolomic data were inconclusive with complications arising from an influence of gender which was not observed with the molecular and behavioural outputs. Nevertheless, the molecular data from the hippocampus of mice administered with a low dose of L-alanine provide a solid basis to further explore L-alanine modulation of asc-1, ASCT2 and Glun2B-NMDARs to improve the treatment of mood disorders.
